# The Role of Nrf2-Mediated Pathway in Cardiac Remodeling and Heart Failure

**DOI:** 10.1155/2014/260429

**Published:** 2014-07-01

**Authors:** Shanshan Zhou, Wanqing Sun, Zhiguo Zhang, Yang Zheng

**Affiliations:** The Cardiovascular Center, The First Hospital of Jilin University, 71 Xinmin Street, Changchun 130021, China

## Abstract

Heart failure (HF) is frequently the consequence of sustained, abnormal neurohormonal, and mechanical stress and remains a leading cause of death worldwide. The key pathophysiological process leading to HF is cardiac remodeling, a term referring to maladaptation to cardiac stress at the molecular, cellular, tissue, and organ levels. HF and many of the conditions that predispose one to HF are associated with oxidative stress. Increased generation of reactive oxygen species (ROS) in the heart can directly lead to increased necrosis and apoptosis of cardiomyocytes which subsequently induce cardiac remodeling and dysfunction. Nuclear factor-erythroid-2- (NF-E2-) related factor 2 (Nrf2) is a transcription factor that controls the basal and inducible expression of a battery of antioxidant genes and other cytoprotective phase II detoxifying enzymes that are ubiquitously expressed in the cardiovascular system. Emerging evidence has revealed that Nrf2 and its target genes are critical regulators of cardiovascular homeostasis via the suppression of oxidative stress, which is the key player in the development and progression of HF. The purpose of this review is to summarize evidence that activation of Nrf2 enhances endogenous antioxidant defenses and counteracts oxidative stress-associated cardiac remodeling and HF.

## 1. Introduction

Despite recent advances in treatment, the morbidity, mortality, and economic burden of heart failure (HF) still remain very high. Hypertension, ischemia, diabetes, and some anticancer drugs used in the clinic are common causes of cardiac remodeling and HF. Cardiac remodeling, a term that refers to cardiac maladaptation at the molecular, cellular, tissue, and organ levels, is the key pathophysiological process leading to HF. It has been well established that oxidative stress is a major cause of HF [1–8].

Free radicals and other reactive small molecules have emerged as important regulators of many physiological and pathological processes [9]. Reactive oxygen species (ROS) and reactive nitrogen species (RNS) serve as signaling messengers to mediate various biological responses [10, 11], including numerous cardiovascular diseases, such as HF, coronary heart disease, and cardiac arrhythmias [12]. Whether the effects of ROS/RNS are beneficial or harmful depends on the site, type, and amount of ROS/RNS production and the activity of the organism's antioxidant defense system [13]. As a rule, heart and cardiovascular diseases are characterized by ROS overproduction, whereas the formation of major RNS, nitric oxide (a free radical) and peroxynitrite (a diamagnetic molecule), can decrease or increase depending on the nature of the heart injury [12]. ROS include superoxide anion (*∙*O^2−^), hydrogen peroxide (H_2_O_2_), hydroxyl radical (*∙*OH), and hypochlorite (OCl^*∙*−^), which impair cardiac function [14] and increase susceptibility to cardiac arrhythmia [15] through direct toxic effect, resulting in increased necrosis and apoptosis of cardiomyocytes [16]. Critical components of the cellular antioxidative defense mechanisms are the ROS scavengers, phase II detoxification enzymes, and other detoxification proteins which contain antioxidant response elements (AREs) in their promoter regions. A major regulator of the AREs is the highly conserved transcription factor, nuclear factor-erythroid-2- (NF-E2-) related factor 2 (Nrf2). Many of the Nrf2-regulated enzymes are essential in the pathogenesis of cardiovascular diseases [17] and are strongly associated with HF; in addition, they serve as sensitive and specific markers to reflect the ventricular function in HF patients [18]. Nrf2 also can prolong graft survival and modulate innate and adaptive immune responses after heart transplantation [19]. Emerging evidence has revealed that the Nrf2/ARE signaling pathway plays an important role in preventing oxidative cardiac cell injury* in vitro* [20, 21] as well as protecting the heart from maladaptive remodeling and cardiac dysfunction [7, 22–27].

The main purpose of this review is to discuss the current evidence for the cardioprotective role of Nrf2 and its target genes in the development of cardiac remodeling and HF caused by hypertension, ischemia, diabetes, and anticancer drugs.

## 2. ROS and Cardiac Remodeling

ROS refer to a group of small reactive molecules that include O2^*·*−^, H_2_O_2_, OH^*·*−^, and OCl^*·*−^. These molecules react avidly with other molecules such as cellular lipids, proteins, and nucleic acids [28]. The fine balance between ROS generated during normal physiological processes and endogenous antioxidants presented in the body is essential for redox homeostasis. Increased production of ROS or impaired endogenous antioxidant defense of the body that leads to oxidative stress may cause adverse effects due to irreversible modification of macromolecules, membranes, proteins, and DNA [29]. Cardiac remodeling is an adaptive, regulatory process of cardiomyocytes that occurs over time in order to maintain homeostasis against external stress [30]. Progression of cardiac hypertrophy, cardiomyocyte apoptosis, and interstitial collagen deposition into systolic dysfunction has been reported in numerous clinical and animal studies [31–35]. ROS can activate specific pathways leading to adaptive or maladaptive cardiac remodeling processes [28]. In experimental studies, oxidative stress has been identified as one of the molecular mechanisms and the key players involved in the development of cardiac hypertrophy [36]. In addition, antioxidants have been found to prevent the development of cardiac hypertrophy [37]. Various studies have suggested that oxidative stress is also an important regulator of profibrotic processes in the myocardium [34, 35]. NADPH oxidase-dependent ROS production contributes to the development of left ventricular (LV) interstitial fibrosis, reduction of ROS generation, and restoration of the redox balance; thus, it may be important in preventing or treating myocardial fibrosis in HF [38]. Programmed cell death (apoptosis) of cardiomyocytes has been identified as an essential process in the progression to HF [39]. ROS and the resulting oxidative stress also play a pivotal role in this pathological process. Antioxidants and thiol reductants, such as N-acetylcysteine, and overexpression of manganese superoxide dismutase (MnSOD) can block or delay apoptosis [40]. Taken together, ROS play an important role in the development of cardiac remodeling and HF. The findings of the majority of the studies suggest that antioxidants may be possible therapeutic candidates against cardiac remodeling.

## 3. The Nrf2-ARE Signaling Pathway

Nrf2 is the master regulator of oxidative stress signaling. It is a member of the cap-n-collar (CNC) family of transcription factors, which include Nrf1-3 and Bach1-2 [41–43]. These genes encode transcription factors that belong to the CNC-type of the basic region leucine zipper factor family (CNC-bZIP). CNC-bZIP factors are characterized by a highly conserved 43-amino acid homology region that lies in the immediate N-terminus of the basic DNA-binding domain and is referred to as the “CNC” domain after the* Drosophila* cap-n-collar protein. There is evidence indicating that CNC-bZIP factors function as obligate heterodimers by forming dimers with small Maf proteins (Maf G, Maf K, and Maf F) for DNA binding [44–46].

**Figure 1:**
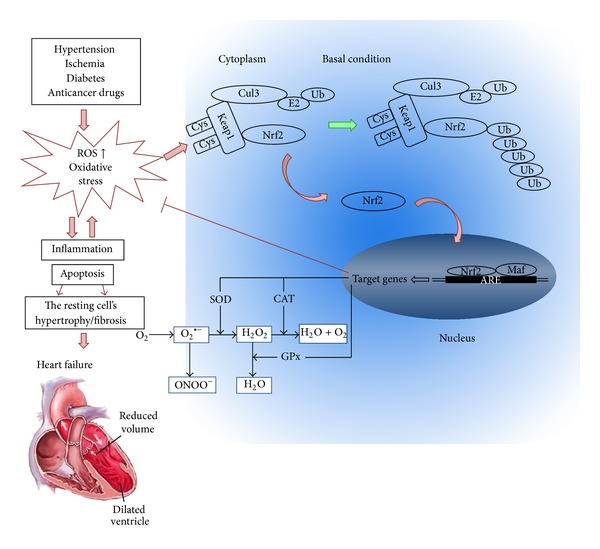
Roles of oxidative stress in cardiac remodeling and the potential protection by Nrf2 from oxidative damage. Hypertension, ischemia, diabetes, and anticancer drugs all induce additional generation of reactive oxygen and/or nitrogen species (ROS and/or RNS), leading to oxidative stress. Oxidative stress accelerates inflammation and apoptosis, which in turn causes cardiomyocyte hypertrophy and/or fibroblast proliferation, resulting in the cardiac remodeling (fibrosis). Meanwhile, ROS and/or RNS interact with cysteine residues in Keap1, disrupting the Keap1-Cul3 ubiquitination system. At the early stage of these pathological conditions, the released Nrf2 from Keap1 translocates to nucleus and combines with Maf and ARE to initiate the transcription of a number of antioxidative genes, such as SOD, CAT, and GPx, which are performing a wide range of cell defense processes against this pathological oxidative stress in the heart; however, at the late stage, Nrf2 may be exhausted or downregulated by its abnormal Nrf2 gene expression, leading to the failure to maintain the redox homeostasis by increasing ARE-mediated expression of phase II and antioxidant enzymes. Consequently, the persistently oxidative stress induces cardiac remodeling and finally heart failure.

Under normal conditions, Nrf2 is kept in the cytoplasm by Kelch-like-ECH-associated protein 1 (Keap1) and Cullin 3 [47]. Cullin 3 ubiquitinates its substrate, Nrf2; and Keap1 serves as a substrate adaptor, which facilitates the ubiquitination of Nrf2 by Cullin 3. As a result, Nrf2 has a short half-life that lasts only 20 min under normal conditions [48]. As illustrated in Figure 1, oxidative stress destroys critical cysteine residues in Keap1, disrupting the Keap1-Cul3 ubiquitination system. If Nrf2 is not ubiquitinated, it builds up in the cytoplasm [49] and is translocated into the nucleus. In the nucleus, Nrf2 combines with a small protein called Maf to form a heterodimer, and, by binding to the ARE in the upstream promoter region, it initiates the transcription of a number of antioxidative genes, including heme oxygenase-1 (HO-1), NAD(P)H dehydrogenase (quinone 1) (NQO1), superoxide dismutases (SODs), catalase (CAT), glutathione-S-transferase (GST), *γ*-glutamylcysteine synthase (*γ*-GCS), and glutathione peroxidase (GPx) [50, 51] (Figure 1). These are the first line of defense mechanism against ROS-mediated cardiac injury. HO-1 is a rapidly inducible cytoprotective protein that degrades heme to biliverdin, ferrous iron, and carbon monoxide (CO) [52]. HO-1 mitigates cellular injury by exerting antioxidant, antiapoptotic, and anti-inflammatory effects [52, 53]. SOD catalyzes the dismutation of O^2−^ into H_2_O_2_ and O_2_. H_2_O_2_ is a product of SOD activity and is handled by GPx and CAT. Three SOD isozymes have been identified, including copper/zinc-containing SOD (CuZn-SOD, also SOD1), which is primarily cytosolic in location, Mn-SOD (also SOD2), and extracellular SOD (EC-SOD, also SOD3) [54]. The GPx/glutathione (GSH) system is important in low-level oxidative stress. It scavenges hydroxyl radicals and singlet oxygen directly, detoxifying H_2_O_2_ and lipid peroxides by the catalytic action of GPx [55]. GPx not only scavenges H_2_O_2_ but also prevents the formation of other more toxic radicals, such as *·*OH [56]. CAT is mainly located in cellular peroxisomes and, to some extent, in the cytosol and catalyzes the reaction of H_2_O_2_ to water and molecular oxygen [57]. Overexpression of mitochondrial CAT has shown protection against cytotoxic drugs [58].

Through inducing the expression of this battery of genes, Nrf2 is able to augment a wide range of cell defense processes, thereby enhancing the overall capacity of cells to detoxify potentially harmful substances. As such, the Nrf2-Keap1 pathway is generally considered to be a major cellular defense pathway. In addition, recent experimental and clinical studies have shown the important roles that Nrf2 and its downstream genes play in the pathogenesis of cardiac remodeling and HF induced by a number of factors.

## 4. Role of the Nrf2 Pathway in Hypertension-Induced Cardiac Remodeling

Many factors are involved in the pathophysiology of hypertension, such as upregulation of the renin-angiotensin-aldosterone system and activation of the sympathetic nervous system [59]. Common to these processes are the increased bioavailability of ROS due to excess ROS generation, decreased nitric oxide level, and reduced antioxidative capacity in the cardiovascular, renal, and nervous systems [60, 61]. In the cardiovascular system, ROS activate a broad variety of hypertrophy-promoting kinases and transcription factors, and oxidative stress has been identified as one of the key contributing factors in the development of cardiac hypertrophy [29]. In a pathologically hypertrophied myocardium, fibrosis and induction of fetal gene expression promote the development of cardiac dysfunction [62–65]. Activation of Nrf2 and its target genes provides a novel mechanism to protect the heart against pathological cardiac remodeling through suppressing oxidative stress [7, 25, 26, 66].

**Figure 2:**
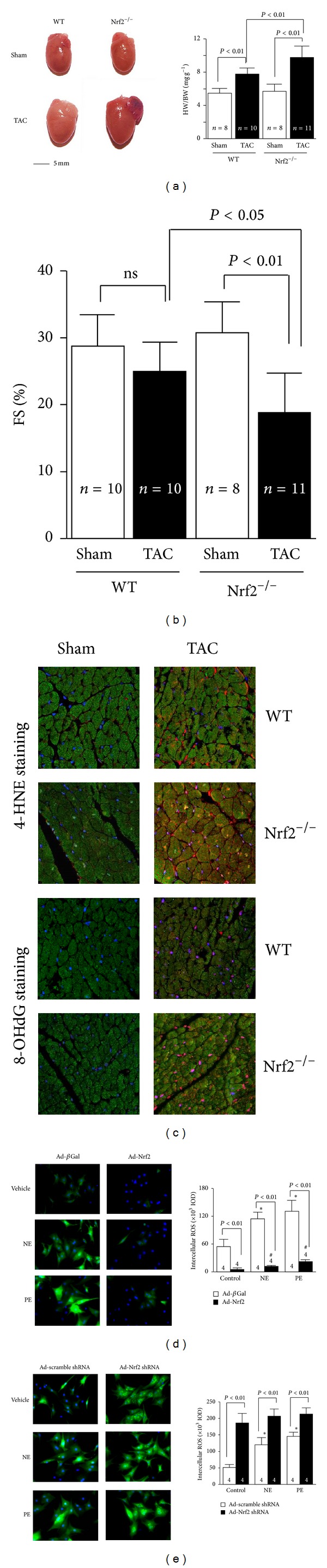
Nrf2 protects against maladaptive cardiac responses to hemodynamic stress. (a) The left panel shows representative pictures of hearts of wild-type (WT) and Nrf2^−/−^ mice after TAC. The right panel shows the heart weight/body weight (HW/BW) ratio. (b) Left ventricle fractional shortening (FS) (%) of WT and Nrf2^−/−^ mice 2 weeks after TAC. (c) Representative confocal microscopic images of LV 4-HNE staining. 4-HNE-positive staining is shown in red. Nuclei are shown in blue (×630). Nrf2 gain- and loss-of-function on hypertrophic factor-induced ROS production in rat neonatal cardiomyocytes. Representative images and quantitative analysis of intracellular ROS production in norepinephrine (NE, 200 *μ*mol/L) or phenylephrine (PE, 100 *μ*mol/L) treated cardiac myocytes that were infected with adenovirus of Nrf2 (d) or rat Nrf2 shRNA (e). *P < 0.05 or ^#^ *P* < 0.05 versus control ad-*β*Gal- or ad-Nrf2-infected cells that were treated with vehicles. The combined figures here were collected by the authors from different figures published in the study by Li et al. [7].

After transverse aortic constriction (TAC), Nrf2 expression was transiently increased and then declined to the basal level [7]. Compared to wild-type mice, Nrf2-knockout mice after TAC developed pathological cardiac hypertrophy, significant myocardial fibrosis and apoptosis, overt HF, and increased mortality, which were associated with elevated myocardial levels of 4-hydroxy-2-nonenal and 8-hydroxydeoxyguanosine and a complete blockade of the myocardial expression of several antioxidant genes. Overexpression of Nrf2 dramatically inhibited hypertrophic factor-induced ROS production and growth in both cardiomyocytes and cardiac fibroblasts, whereas knockdown of Nrf2 exerted the opposite effects in both cells [7]. Thus, Nrf2 is a critical regulator for maintaining structural and functional integrity of the heart that is abnormally stressed, as shown in Figure 2. Activating transcription factor 3 (ATF3) is a cAMP response element-binding protein/ATF family transcription factors member and has been implicated in the cardiovascular and inflammatory systems [67]. Infusion of tert-butylhydroquinone (tBHQ), a selective ATF3 inducer, increased the expression of ATF3 via the Nrf2-related transcriptional factor, inhibited transverse aortic banding-induced cardiac dilatation, and increased LV contractility, thereby rescuing from HF [67].

In addition, HO-1 protects against cardiac hypertrophy [24, 25, 68]. For example, Hu et al. reported that HO-1 attenuated angiotensin II- (Ang II-) induced cardiac hypertrophy both* in vitro* and* in vivo* [25]. Furthermore, Ndisang and Jadhav reported that upregulating the heme oxygenase system suppressed LV hypertrophy in spontaneously hypertensive adult rats and was accompanied with attenuated extracellular matrix remodeling, whereas HO-1 was blocked with chromium mesoporphyrin-exacerbated cardiac fibrosis/hypertrophy [68]. Deletion of Bach1 caused upregulation of cytoprotective HO-1 and also inhibited TAC-induced LV hypertrophy and remodeling [69]. Another group reported that HO-1 gene transfer to cardiomyocytes attenuated Ang II-mediated apoptosis but not hypertrophy [70]. However, Chen et al. reported that, after TAC, more calcineurin protein expression was induced in systemic HO-1 transgenic overexpressing mice than in wild-type mice, and it aggravated pressure overload-induced cardiac hypertrophy [71]. Therefore, the role of HO-1 in pressure overload-induced cardiac remodeling is controversial and further investigation is needed.

Myocardial SOD activity was suppressed in LV remodeling induced by TAC [26, 72]. In addition, Ang II could stimulate collagen production and in the meantime inhibit total SOD, SOD1, and SOD2 activity in rat cardiac fibroblasts [73]. Reduced expression of the manganese-dependent SOD2 resulted in increased cardiac oxidative stress and induced maladaptive cardiac hypertrophy [74]. Moreover, SOD3 deficiency exacerbated TAC-induced myocardial oxidative stress, hypertrophy, fibrosis, and HF [26]. These findings demonstrate that activation of Nrf2 and its downstream genes provides a novel mechanism to protect the heart against pressure overload-induced pathological cardiac hypertrophy, fibrosis, apoptosis, and HF via suppressing oxidative stress.

## 5. Role of the Nrf2 Pathway in Ischemia-Induced Cardiac Remodeling

Coronary artery disease and ischemic heart disease are prevalent worldwide. Cardiac hypertrophy, apoptosis, and fibrosis after myocardial infarction (MI) have been identified as key detrimental factors in the development of HF. The development of percutaneous coronary intervention and surgical revascularization has brought marked benefits to patients with acute MI. However, ischemia/reperfusion (I/R) injury during revascularization can cause further cardiac injury [75, 76]. Once blood flow is restored, ROS can be produced either by the myocardium itself or by infiltrating inflammatory cells [77]. ROS can subsequently lead to cellular damage through a number of pathways, including direct damage to membranes and proteins or indirect damage through activation of proapoptotic pathways [77]. This damage can further cause cardiac remodeling which leads to progressive heart chamber dilation, ventricular wall thinning, and eventually HF [78]. Nrf2 and its target genes have been shown to play a protective role in cardiac ischemia-associated injury [79, 80].

Some antioxidants protect the heart from ischemia-induced cardiac injury via the Nrf2 pathway. For example, *α*-lipoic acid and prostaglandin D2 significantly increased Nrf2 nuclear translocation and the expression of its downstream genes reduced lactate dehydrogenase (LDH) and creatine kinase (CK) release, attenuated myocardial infarct size, decreased cardiomyocyte apoptosis, and partially preserved heart function; and this effect was at least partially PI3K/Akt signaling pathway dependent [79, 81]. The phenomenon of ischemic preconditioning (IPC) has been recognized as one of the most potent mechanisms to protect against myocardial ischemic injury, reducing infarct size, attenuating the incidence and severity of reperfusion-induced arrhythmias, and preventing endothelial cell dysfunction [82]. In rat hearts, 30 minutes of left anterior descending coronary artery occlusion resulted in a reduction in the Nrf2 protein level, which was prevented by IPC of the myocardium [83]. Recently, Zhang et al. [23] reported IPC-induced activation of protein kinase C, which then promoted the translocation of Nrf2 and the inductions of antioxidant genes HO-1 and MnSOD and decreased tissue malondialdehyde content compared to control hearts.

Prior studies have reported that HO-1 is upregulated in human failing hearts [84] and in animal models of right ventricular failure [85]. The HO-1 expression in the noninfarct myocardium was increased four weeks after coronary ligation, and cardiomyocyte-specific HO-1 transgenic mice showed improved postinfarction survival and attenuated cardiac hypertrophy, interstitial fibrosis, oxidative stress, and apoptosis [86, 87]. Heterozygous HO-1^+/−^ mice exhibited exaggerated cardiac injury and dysfunction after I/R, patially rescued by antioxidants [88]. In contrast, mice with cardiac-restricted HO-1 overexpression were resistant to I/R injury, with improved contractile recovery and reduced infarct size, inflammatory cell infiltration, oxidative damage, and apoptosis [89]. Similar results were obtained in rat hearts subjected to I/R 8 weeks after human HO-1 gene transfer [90]; the improvement in LV function was maintained for up to 1 year after injury [91]. Kuzuya et al. [92, 93] have shown that the infarct limitation observed in the canine myocardium 24 hours after IPC was accompanied by a significant increase in SOD2 activity. In addition, a recent study demonstrated that SOD3 expression was decreased in MI-induced HF [54]. SOD3-knockout mice had greater increases of nitrotyrosine in the peri-infarct myocardium, and this was associated with a greater reduction of LV ejection fraction, a greater decrease of sarcoplasmic or endoplasmic reticulum Calcium^2+^ ATPase, and a greater increase of atrial natriuretic peptide in the peri-infarct zone compared to wild-type mice at 8 weeks after MI, which means that mice with SOD3 gene deletion developed more severe LV hypertrophy, more fibrosis, and worse cardiac function [54]. Moreover, it has been demonstrated that GPx-knockout mice were more susceptible to myocardial I/R injury and transgenic mice were more resistant to myocardial I/R injury [94–96]. Overexpression of GPx also attenuated post-MI cardiac failure and cardiac remodeling by decreasing myocyte hypertrophy, apoptosis, and interstitial fibrosis in the noninfarct LV [56], which may be related to GPx preserving electron transport chain complex activities [96]. Overexpression of Nrf2 and its downstream genes inhibited ischemia-induced LV remodeling and HF; thus, therapies designed to interfere with oxidative stress might be beneficial to prevent ischemia-induced heart injury.

## 6. Role of the Nrf2 Pathway in Diabetes-Induced Cardiac Remodeling

Diabetic cardiomyopathy is a very common complication of diabetes and also one of the major causes of mortality in diabetic patients [97]. Evidence suggests that diabetes is associated with a reduced overall antioxidant defense system and increased oxidative stress [98]. Extra production of ROS and RNS causes the development of diabetic complications, including cardiomyopathy; on the other hand, antioxidants can prevent or treat diabetic complications [97, 99, 100]. Therefore, the activation of endogenous antioxidative components has been proposed as an appealing strategy to alleviate diabetic complications [101]. Nrf2 and its target genes may prevent or inhibit this pathological process through their antioxidative stress properties.

Emerging evidence has indicated that high glucose (HG) not only induces ROS and/or RNS production, but also enhances the expression and activation of Nrf2 and its downstream genes, both* in vivo* and* in vitro* [103, 115]. However, Tan et al. have reported a different finding [116]. In tissue sections of left ventricles obtained from autopsied heart specimens of humans with or without diabetes, Nrf2 expression in the nuclei was significantly downregulated compared to control hearts [116]. Tan et al. also demonstrated that Nrf2 protein expression was slightly increased in the hearts of mice with two-month hyperglycemia but significantly decreased in the hearts of mice with five-month hyperglycemia [116], which suggests that Nrf2 was adaptively overexpressed to combat diabetic damages at the early stage of diabetes; at the late stage, however, cardiac antioxidant function was so severely impaired that it led to a decrease in cardiac Nrf2 expression [117]. Glucose at high concentrations induced ROS production in both primary neonatal and adult cardiomyocytes from the Nrf2 wild-type mouse heart, whereas, in Nrf2-knockout cells, the amount of ROS was significantly greater under basal conditions and high glucose markedly further increased ROS production in concentration- and time-dependent manners. Concomitantly, high glucose induced significantly greater levels of apoptosis at lower concentrations and in a shorter time in Nrf2-knockout cells than in wild-type cells [115].

HO-1 and its reaction products have been shown to have both antioxidative and anti-inflammatory properties [89]. Association of a microsatellite polymorphism in the promoter region of the human HO-1 gene with the risk of coronary artery disease in type 2 diabetic patients has been reported [118]. Patients with type 2 diabetes with longer repeats of the HO-1 gene promoter (with lower HO-1 inducibility) were shown to have more oxidative stress and increased susceptibility to coronary artery disease [118]. In streptozotocin- (STZ-) induced diabetes rodent models, overexpression of HO-1 ameliorated LV dysfunction, myofibril structure disarray, oxidative stress, inflammation, apoptosis, and autophagy [119]. Furthermore, deficiency of HO-1 significantly increased the infarct size in normoglycemic mice and exacerbated myocardial infarction in diabetic mice [120]. In addition to a larger infarct size, mortality was two-fold greater in diabetic HO-1-knockout mice than in wild-type mice after I/R injury, and 55% of the diabetic HO-1-knockout mice survived LV thrombi induced by I/R [120]. Myocardial SOD activity and the GSH level were also significantly increased in the hearts of diabetic rats [114]. Overexpression of SOD2 protected the cardiac morphological changes induced by diabetes and completely normalized the impaired contractility in diabetic cardiomyocytes [121]. Moreover, the plasma type of GPx, GPx-3, was significantly upregulated in diabetic mice compared with control mice [122]. Furthermore, GPx overexpression inhibited the development of LV remodeling and diastolic dysfunction associated with diabetes [27]. These beneficial effects of GPx overexpression are thought to be associated with the attenuation of hypertrophy, apoptosis, and interstitial fibrosis of cardiomyocytes [27]. Thus, Nrf2 and its downstream genes play an essential protective role in the adaptation of diabetic cardiomyopathy. Taken together, these studies may provide a new target for developing therapeutic strategies to treat/prevent diabetic cardiomyopathy.

## 7. Role of the Nrf2 Pathway in Anticancer Drug-Induced Cardiac Remodeling

There are rising concerns among both cardiologists and oncologists about cardiotoxicity induced by cancer treatment, since it has a significant impact on the management and outcomes of cancer patients. The most typical manifestation of cardiotoxicity is hypokinetic cardiomyopathy leading to HF [123]. Nrf2 and its target genes have been suggested to be useful for protection against cardiotoxicity of anticancer drugs [124, 125].

Anthracycline anticancer drugs (e.g., doxorubicin or daunorubicin) can induce chronic cardiotoxicity and HF, both of which are believed to be caused by oxidative injury and mitochondrial damage [126]. GPx activity was significantly increased in both a daunorubicin-induced rabbit HF model and rat cardiomyocytes exposed to daunorubicin [127]. Incubation of H9c2 rat cardiomyoblasts with doxorubicin resulted in a two-fold increase in Nrf2 protein content and enhanced transcription of several of the Nrf2-regulated downstream genes, including GSTp1 and NQO1 [128]. Chronic arsenic exposure also has been linked to increased risks of vascular diseases. Arsenic exposure affects the activity of Nrf2 [125], and HO-1 has been identified as a response biomarker [129]. H9c2 rat cardiomyocytes exposed to arsenic showed a modest activation of Nrf2 and lower GSH availability [130]. However, the results have been contrastive. Recently, in a daunorubicin-induced rabbit HF model, Jirkovský et al. showed that Nrf2 and its target genes did not show upregulation and that global oxidative stress may not be a factor for the development of anthracycline-induced HF [131]. Therefore, the role of Nrf2 and its target genes in anticancer drug-induced cardiotoxicity is still controversial and more investigations are needed.

## 8. Potential Clinical Interventions of Cardiac Remodeling by Targeting Nrf2 and Its Target Genes

The exploration of mechanisms underlying the regulation of the Nrf2 pathway has led to the development of agents manipulating Nrf2 to treat HF. In fact, compounds that increase the activity of Nrf2 and its downstream genes are currently being tested for disease prevention [111, 132–134] (Table 1).

### 8.1. Activation of Nrf2 to Treat Cardiac Remodeling and HF

Many Nrf2 activators are natural products and plant-derived phytochemicals. Some examples of natural Nrf2 activators include sulforaphane, curcumin, resveratrol, and garlic organosulfur compounds [135]. Several synthetic Nrf2 activators have also been developed, such as carbobenzoxy-Leu-Leu-leucinal (MG132), 4-hydroxynonenal (4-HNE), *α*-lipoic acid, hydrogen sulfate, and 17*β*-estradiol (E2) [79, 109, 110]. These chemopreventive compounds exert their effects by inducing an Nrf2-mediated defense response as well as activation of phase II detoxification enzymes, antioxidants, and transporters that protect cells from oxidative stress.

#### 8.1.1. Natural Nrf2 Activators

One of the most extensively studied natural products that target the Nrf2-Keap1 signaling pathway is sulforaphane, an isothiocyanate present in cruciferous vegetables such as broccoli [136]. Treatment with sulforaphane decreased infarct size, inhibited an increase in the LV end-diastolic pressure, and improved coronary flow in mice after MI [102]. This protective effect may be partly mediated through HO-1, SOD, and CAT expression [102].

Curcumin is another well-studied chemopreventive natural product that is capable of activating Nrf2 [132]. Curcumin has been used to attenuate acute doxorubicin-induced cardiomyopathy in rats [133]. In this study, curcumin pretreatment reversed the increase in lipid peroxidation and CAT content, as well as the decrease in GSH content and GPx activity in rat cardiac tissues induced by doxorubicin [133].

By treating rat cardiac stem cells with resveratrol in a rat left anterior descending occlusion model, Gorbunov et al. found that resveratrol significantly improved cardiac function through enhancing Nrf2 expression, as well as significantly increasing the survival and engraftment of implanted cardiac stem cells in the host [134]. Resveratrol can also achieve the same effect by gavage in the same animal model [107].

In an Ang II-induced cardiac remodeling and HF rat model, allicin treatment could prevent the development of cardiac remodeling and the progression of cardiac hypertrophy to cardiac dysfunction, by enhancing the Nrf2 pathway [108]. In addition, dietary phytochemical intake could upregulate the cardiac Nrf2 transcriptome and reduce oxidative damage and HF in hypertensive rats [137]. The protective effect of Nrf2 in myocardial remodeling and HF has been suggested to be mediated through Nox4 [138], which is known to be an important regulator of reduction-oxidation signaling in many cell types including cardiomyocytes [139].

#### 8.1.2. Synthetic Nrf2 Activators

Accumulating evidence has demonstrated that MG132 can protect cells and tissues against oxidative damage, since it can activate the Nrf2-ARE signaling pathway, leading to upregulation of detoxifying and antioxidant genes [140, 141]. Both sulforaphane and MG132 could prevent diabetes-induced high blood pressure and cardiac dysfunction, as well as cardiac hypertrophy, fibrosis, oxidative damage, and inflammation [103, 106]. In pressure-overload-induced rodent HF models, treatment with MG132 significantly attenuated cardiac hypertrophy and cardiac fibrosis as well as improving cardiac function [104, 105]. Mechanistically, MG132 may enhance Nrf2-mediated antioxidative function and inhibit NF-*κ*B-mediated inflammation [142].

4-HNE is an *α*, *β*-unsaturated hydroxyalkenal that is produced by lipid peroxidation in cells. *α*-Lipoic acid is an organosulfur compound derived from octanoic acid. E2 is a sex hormone. All three of these compounds could protect the heart from ischemia-induced cardiac remodeling and HF by activating Nrf2 and its target genes. Treatment with 4-HNE [110], *α*-lipoic acid [79], or hydrogen sulfate [109] could activate Nrf2 in the heart and increase the intramyocardial GSH content, consequently improving the functional recovery of the LV following I/R in Langendorff-perfused hearts [110] or reducing infarct size, decreasing cardiomyocyte apoptosis* in vivo* [79, 109]. The cardioprotective effect of 4-HNE was not observed for Nrf2-knockout mice [110], indicating that this protection is Nrf2 dependent. In an* in vitro* study, Yu et al. cultured primary cardiomyocytes and established a hypoxia/reoxygenation (H/R) model to simulate myocardial I/R injury [143]. They found that E2 could upregulate Nrf2 expression in nuclear extracts and also increased the expression of HO-1, SOD1, GST, and glutamate-cysteine ligase (GCL) dramatically during H/R injury [143]. These findings indicate that Nrf2 plays a pivotal role in preventing and alleviating cardiac I/R injury-induced oxidative stress.

### 8.2. Role of Nrf2 Target Genes Activation in Cardiac Remodeling

#### 8.2.1. Role of HO-1 Activation in Cardiac Remodeling

It has been reported that elevation of HO-1 by treatment with cobalt protoporphyrin IX (CoPP IX) or a recombinant adenovirus carrying the human HO-1 gene attenuated cardiac hypertrophy and apoptosis, in both an Ang-II-induced HF model and a spontaneously hypertensive rat model [25, 70], while this pathological process was exacerbated in the presence of tin protoporphyrin, an inhibitor of HO activity [70]. In a spontaneous hypertensive rat model, HO-1 upregulation by CoPP IX administration decreased blood pressure, inhibited inflammation, and improved the ventricular remodeling process and postinfarct cardiac function [111]. In addition, cardiac stem cells (CSCs) were protected by pretreatment with CoPP against apoptosis through activation of the extracellular signal-regulated kinase (ERK)/Nrf2 signaling pathway and cytokine release [144].

Hemin could upregulate HO activity, reduce hypertension, suppress oxidative stress, and attenuate cardiac fibrosis, apoptosis, and oxidative stress. This effect was modulated through enhanced expression of the PI3K p85 regulatory subunit [145]. In an acute I/R with STZ-induced hyperglycemic rat model, intraperitoneal administration of hemin 18 h before I/R increased the levels of HO-1, providing marked protection against myocardial injury [146]. Moreover, zinc protoporphyrin IX (an inhibitor of HO activity) abolished the protective effect by hemin [146]. Furthermore, chronic HO-1 activation by prolonged administration of hemin improved postinfarction survival and exerted protective effects in a rat model of myocardial ischemia, through a potent antioxidant activity [112].

Taurine treatment significantly improved LV systolic and diastolic function in an STZ-induced diabetes rat model, and there were persistent increases in activities of Akt/PKB and SOD, as well as the level of HO-1 protein [147].

Mesenchymal stem cells (MSCs) have been reported to have the potential to release several kinds of cytokines, which could induce angiogenesis [148, 149]. However, almost all transplanted cells seemed to be lost by 6 weeks after transplantation, making it impossible for the limited number of MSCs to achieve a maximum proangiogenesis effect [150]. Due to its multiple catalytic byproducts, HO-1 has been proposed to be involved in a number of cytoprotective effects. HO-1 has been administered to improve the survival environment of MSCs and to achieve maximum functional benefits of MSCs [151]. HO-1 transduced by MSCs can induce angiogenic effects, reduce cardiac remodeling, and improve heart function after acute MI [152, 153]. Intramyocardial delivery of the HO-1 gene using adeno-associated virus-2 (AAV-2) before I/R also prevented cardiomyocyte apoptosis and reduced infarct size and cardiac dysfunction after myocardial I/R in mice [90]. To evaluate the long-term effects of HO-1 gene delivery, Liu et al. have showed that delivery of the HO-1 gene reduced mortality and preserved LV function and chamber dimensions 1 year after acute MI [91]. These results suggest that preemptive HO-1 gene delivery may be useful as a therapeutic strategy to reduce post-MI LV remodeling and HF.

#### 8.2.2. Role of SOD and GSH Activation in Cardiac Remodeling

EUK-8, as a SOD mimetic, improved LV end-systolic dimensions and fractional shortening, prevented myocardial oxidant stress, attenuated necrotic and apoptotic cell death, and attenuated cardiac hypertrophy and fibrosis in mice subjected to pressure overload [113].* In vitro*, the SOD mimetics tempol and EUK-8 could also reduce collagen production in Ang-II-treated fibroblasts [73]. Moreover, N-acetylcysteine, an antioxidant and GSH precursor, attenuated diabetic myocardial dysfunction via upregulating myocardial GSH and SOD2 activity [114]. Treatment with polyethylene glycol-conjugated SOD ameliorated doxorubicin-induced cardiac dysfunction, and this effect was mediated by inhibition of doxorubicin-induced upregulation of NF-*κ*B signaling, lowering the levels of hexanoyl-lysine (a marker of free radical-induced lipid peroxidation) and suppressing the activation of Akt and Akt-regulated gene expression [124].

Recombinant SOD3 preserved cardiac function following I/R in isolated rat hearts [154–156] and reduced the infarct size when given just prior to coronary reperfusion in pigs [157]. Cardiac-selective expression of SOD3 from the cardiac troponin-T promoter after systemic administration of AAV-9 provided significant protection against both acute MI and LV remodeling [158]. Liu et al. also have demonstrated that encapsulation of CSCs in SOD-loaded alginate hydrogel enhanced CSC survival in the presence of doxorubicin, indicating its potential application as a novel therapy for the treatment of acute and early-onset doxorubicin-induced cardiotoxicity [159].

## 9. Advantages of Nrf2 Activation in Cardiac Remodeling and HF

Nrf2 dimerizes with members of the small Maf family to bind to antioxidant or electrophile response elements (AREs/EpREs) located in the regulatory regions of cellular defense enzyme genes [50]. The target genes of Nrf2 include many antioxidant genes, such as HO-1, NQO1, SODs, CAT, GST, *γ*-GCS, and GPx. By inducing the expression of these genes, Nrf2 activates a wide range of cell defense processes, thereby enhancing the overall capacity of cells to detoxify and eliminate harmful substances. As mentioned above, activated Nrf2 and its target genes, such as HO-1, SOD, and GPx, all play important roles in preventing cardiac remodeling. Although it cannot be excluded that some Nrf2 target genes may protect the heart through an Nrf2-independent pathway, Nrf2-induced cell defense processes remain to be the main driving force. Because Nrf2 could induce transcriptional activation of a number of ARE-bearing antioxidants, we speculate that, acting as an upstream gene, activation of Nrf2 may rescue from cardiac remodeling and HF induced by deficiency of an individual downstream gene. So, activation of Nrf2 may have an advantage over its downstream target genes in protecting one from oxidative stress-induced HF, but further investigations are needed before a conclusion can be drawn.

## 10. Conclusions

Increased generation of ROS in cardiomyocytes leads to increased necrosis and apoptosis, which induce cardiac remodeling and dysfunction. Nrf2 and its target genes are key components to maintain cellular redox homeostasis by attenuating oxidative stress-associated pathological processes. Patients with insufficient Nrf2 levels in their cardiovascular system are more vulnerable to HF development. It is conceivable that Nrf2 orchestrates a group of antioxidative and other cytoprotective genes to provide a protective mechanism against detrimental stress-induced cardiac dysfunction. However, further work is needed to understand the role of Nrf2 in the pathogenesis of cardiac remodeling and HF in more detail before it can be seriously considered as a therapeutic target for HF.

## Figures and Tables

**Table 1:** Activation of Nrf2 and its downstream genes to treat cardiac remodeling and HF.

	Name	Target disease model	Species	Function	Ways and volume	References
Nrf2 activators	Sulforaphane	Ischemia (I/R, Langendorff)	SD rats	Prevent cardiac apoptosis	0.5 mg/kg/d, 3 days, IP	[102]
		Diabetes (type 1, STZ)	Mice	Prevent cardiac hypertrophy, fibrosis, and apoptosis	0.5 mg/kg/d, 3 months, IP	[103]
	MG132	Hypertension (abdominal aortic banding)	SD rats	Prevent cardiac hypertrophy and fibrosis	0.1 mg/kg/d, 8 or 16 weeks, IP	[104, 105]
		Diabetes (type 1, OVE)	Mice	Prevent cardiac hypertrophy and apoptosis	10 *μ*g/kg/d, 3 months, IP	[106]
	Resveratrol	Ischemia (MI, LAD occlusion)	SD rats	Improve cardiac stem cells' survival, proliferation, and differentiation	2.5 mg/kg/d, 2 weeks, gavage	[107]
	Allicin	Hypertension (Ang II)	SD rats	Prevent cardiac hypertrophy and fibrosis	180 mg/kg/d, 8 weeks, receiving a diet in addition to standard chow	[108]
	*α*-Lipoic acid	Ischemia (I/R, coronary artery ligation followed by reperfusion)	SD rats	Prevent cardiac apoptosis	15 mg/kg, one time, IV	[79]
	Hydrogen sulfate	Ischemia (I/R, coronary artery ligation followed by reperfusion)	Mice	Prevent cardiac apoptosis	0.1 mg/kg, one time, IV	[109]
	4-HNE	Ischemia (MI, Langendorff)	Mice	Improve cardiac function	4 mg/kg, one time, IV	[110]
HO-1 activators	CoPP	Hypertension (Ang II)	Wistar rats	Prevent cardiac hypertrophy	1 mg/kg, every 2 days, 2 weeks, IP	[25]
		Hypertension plus ischemia (spontaneous hypertensive rats + LAD occlusion)	Wistar rats	Prevent cardiac hypertrophy	4.5 mg/kg, one/week, 6 weeks, IP	[111]
	Hemin	Ischemia (MI, LAD occlusion)	SD rats	Prevent cardiac apoptosis	4 mg/kg, every 2 days, 4 weeks, IP	[112]
SOD activators	EUK-8	Hypertension (transverse aortic banding)	Mice	Prevent cardiac hypertrophy, fibrosis, and apoptosis	25 mg/kg/d, 4 weeks, IP	[113]
	N-Acetylcysteine	Diabetes (STZ)	Wistar rats	Improve cardiac function	1.4-1.5 g/kg/d, 8 weeks, in drinking water	[114]

## Abbreviations

HF: Heart failure
ROS: Reactive oxygen species
RNS: Reactive nitrogen species
Nrf2: Nuclear factor-erythroid-2- (NF-E2-) related factor 2
LV: Left ventricular
AREs: Antioxidant response elements
CNC: Cap-n-collar
CNC-bZIP: CNC type of basic region leucine zipper
Keap1: Kelch-like-ECH-associated protein 1
HO-1: Heme oxygenase-1
NQO1: NAD(P)H dehydrogenase (quinone 1)
SODs: Superoxide dismutases
CAT: Catalase
GST: Glutathione-S-transferases
*γ*-GCS: *γ*-Glutamylcysteine synthase
GPx: Glutathione peroxidases
TAC: Transverse aortic constriction
STZ: Streptozotocin
ATF3: Activating transcription factor 3
LDH: Lactate dehydrogenase
GCL: Glutamate-cysteine ligase
GSH: Glutathione
MG132: Carbobenzoxy-Leu-Leu-leucinal
4-HNE: 4-Hydroxynonenal
E2: 17*β*-Estradiol
H/R: Hypoxia/reoxygenation
CSCs: Cardiac stem cells
ERK: Extracellular signal-regulated kinase
MSCs: Mesenchymal stem cells
AAV-2: Adeno-associated virus-2
EpREs: Electrophile response elements
*∙*O^2−^: Superoxide anion
*∙*OH: Hydroxyl radical
H_2_O_2_: Hydrogen peroxide
OCl^*∙*−^: Hypochlorite
Ang II: Angiotensin II
MI: Myocardial infarction
I/R: Ischemia/reperfusion
HG: High glucose
tBHQ: Tert-Butylhydroquinone
CK: Creatine kinase.
